# A Novel Prognostic Model Incorporating Carcinoembryonic Antigen in 3-Week or Longer Postoperative Period for Stage III Colon Cancer: A Multicenter Retrospective Study

**DOI:** 10.3389/fonc.2020.566784

**Published:** 2020-12-01

**Authors:** Jin Fan, Yanlong Liu, Xin Cai, Jingwen Wang, Rui Guo, Yuan Ji, Chao Li, Ye Xu, Xinxiang Li, Chundong Zhang, Rui Zhang, Ji Zhu, Sanjun Cai

**Affiliations:** ^1^ Department of Radiation Oncology, Fudan University Shanghai Cancer Center, Shanghai, China; ^2^ Department of Oncology, Shanghai Medical College of Fudan University, Shanghai, China; ^3^ Department of Colorectal Surgery, Harbin Medical University Cancer Hospital, Harbin, China; ^4^ Department of Radiation Oncology, Shanghai Proton and Heavy Ion Center, Shanghai, China; ^5^ Department of Colorectal Surgery, Cancer Hospital of China Medical University, Liaoning Cancer Hospital & Institute, Shenyang, China; ^6^ Department of Public Health Sciences, The University of Chicago, Chicago, IL, United States; ^7^ Department of Radiation Oncology, Huashan Hospital Fudan University, Shanghai, China; ^8^ Department of Colorectal Surgery, Fudan University Shanghai Cancer Center, Shanghai, China; ^9^ Department of Gastrointestinal Surgery, The Fourth Affiliated Hospital of China Medical University, Shenyang, China; ^10^ Department of Gastrointestinal Surgery, Graduate School of Medicine, The University of Tokyo, Tokyo, Japan

**Keywords:** carcinoembryonic antigen, colon cancer, prognostic factors, tumor-node-metastasis staging, disease-free survival

## Abstract

**Background:**

The prognostic stratification of colon cancer using only the tumor-node-metastasis (TNM) stage has some limitations. We sought to increase the accuracy of stratifying patients with stage III colon cancer by constructing a prognostic model combining carcinoembryonic antigen (CEA) with TNM.

**Methods:**

We retrospectively analyzed the data generated from stage III colon cancer patients who had early postoperative CEA measurement from 21 to 100 days after surgery from 2006 to 2017. CEA value was processed using restricted cubic splines (RCS) method. The prognostic model was developed using cox proportional hazards regression.

**Results:**

The time later than 20 days after surgery was optimal for measuring CEA, which was determined by comparing the prognostic value for preoperative and postoperative CEA (N = 2,049), and by evaluating the relationship between the hazard ratio (HR) and postoperative CEA measuring time. Postoperative CEA, T stage and N stage were selected into the final model, and the mean integrated-AUC (iAUC) was 0.78 with 1,000 × bootstrap resampling, which was higher than the model using only T and N stages (TN model; mean iAUC, 0.66). The net reclassification improvement (NRI) was 15% when compared with TN model. Patients could be divided into high and low risk groups by the model, and 3-year disease-free survival (DFS) were 53.7% and 87.0%, respectively (HR, 4.30; 95% CI, 2.65 to 6.96; P < 0.001). Similar results were found in the validation set.

**Conclusions:**

Stage III colon cancer could be stratified more accurately using the new prognostic model combining postoperative CEA with T and N stage.

## Introduction

The prognosis of colorectal cancer is classified using the AJCC/UICC TNM classification system, which has been increasingly challenged in recent years. A large-sample analysis based on the Surveillance, Epidemiology, and End Results (SEER) database showed that the 5-year survival rate of patients with T1-2N1 colon cancer (71.1%) was similar to that of patients with T3N0 disease (66.7%) (1), indicating that the prognosis of some stage III colon cancer patients is similar to that of stage II patients according to this staging system. The population of patients with stage III colon cancer is a heterogeneous group with varying prognoses that cannot be adequately distinguished by the TNM classification system. Unfortunately, these patients were subjected to the same treatment regimens, specified in the NCCN or ESMO guidelines until the findings from the International Duration Evaluation of Adjuvant Chemotherapy (IDEA) study were published (2). The IDEA study not only indicated that high and low risk stratification is necessary but also suggested the need to identify a more appropriate prognostic biomarker.

Carcinoembryonic antigen (CEA) is an effective serum biomarker that is recommended as part of the preoperative work-up and postoperative follow-up routine in patients with colorectal cancer. Multiple studies have demonstrated the usefulness of preoperative CEA for predicting the prognosis of colon cancer patients (3–5), while other studies have suggested that postoperative CEA performed better than preoperative CEA in prognostic stratification; moreover, the recurrence-free survival of patients with an elevated preoperative CEA level but a normalized postoperative CEA level was not significantly different from that of patients with a normal preoperative CEA (6). In the present study, we aimed to determine whether preoperative or postoperative serum CEA could be used to optimize the current prognostic model in patients with stage III colon cancer.

## Material and Methods

### Study Design and Patients

We firstly assessed the relationship pattern between postoperative CEA (within 100 days after surgery) and disease-free survival (DFS), explored the appropriate measurement time for CEA and determined the optimal method for CEA data processing. We further combined postoperative CEA with other clinical factors (such as TNM staging), to build a better prognostic model for stage III colon cancer. Patients were then divided into high-risk and low-risk groups according to this new model.

A total of 9,893 patients with stage I–III colorectal cancer underwent curative resection at the Fudan University Shanghai Cancer Center (FUSCC, Shanghai, China), between January 2006 and January 2017. To build the final model, we included the patients who met all of following criteria: (1) stage III colon cancer; (2) received curative resection; (3) with the CEA record during 21–100 day after resection; and (4) with available follow-up information. Patients were excluded if they received preoperative chemotherapy or radiotherapy or were confirmed with metastasis before or during surgery. A validation set was collected from two high-volume cancer centers, Liaoning Cancer Hospital (Shenyang, China) and Harbin Medical University Cancer Hospital (Harbin, China). Data was obtained with institutional review board approval.

### Management, Surveillance, and Outcome

All patients were restaged according to the UICC/AJCC 8th TNM classification (7). Adjuvant chemotherapy had been administered to patients with stage III or high-risk stage II disease following pathological evaluation of the surgical specimen, as recommended in the NCCN guidelines. Postoperative surveillance of stage I–III colorectal cancer had been also performed according to the national guidelines. Follow-up data was collected by reviewing medical records (including radiographic reports) or through telephone.

The primary outcome was DFS, which was calculated from the date of resection until the date of recurrence, metastasis, or death attributable to any causes which came first. Patients who did not experience any of these events during follow-up were censored at the last follow-up.

### Statistical Analysis

We performed statistical analyses using R version 3.5.1. CEA value was processed using restricted cubic splines (RCS) and dichotomization method respectively (8). DFS was estimated using the Kaplan-Meier method, and groups were compared using a log-rank test. The Cox proportional hazards assumption was tested. A Cox proportional hazards regression model was used for univariate and multivariate modeling and to examine the prognostic significance of the variables identified in the models. Variables with values of *P* < 0.10 from the univariate analysis were then included in the multivariate analysis. Backward stepwise selection was used to obtain the final multivariate model, and variables with values of *P* < 0.05 were retained in the final model.

The predictive accuracy of the model was evaluated using integrated-AUC (iAUC, the integrated value of time-dependent AUC) (9, 10). Accordingly, 1,000 × bootstrap resampling validation was used for unbiased estimation. Model performance was compared also using net reclassification improvement (NRI) (11, 12), decision curve analysis (DCA) (13–15) and likelihood ratio test. Individual-based probability improvement was calculated by the difference of probability bias for each patient (probability bias = predicted survival rate − actual survival rate; probability improvement: probability bias for new model minus probability bias for standard mode). The relative importance of each parameter with respect to survival risk was assessed using the χ² from Harrell’s rms R package. An interactive web-tool based on the model was developed, which was more convenient than nomogram. According to predicted 3-year DFS and using 75% as cutoff-point, high-risk and low-risk groups was divided, because the 3-year DFS of patients with stage III colon cancer was approximately 75% in the FUSCC database and IDEA study (2). Other series potential cut-points were also considered to evaluate the stratification capability of the model.

## Results

### Postoperative CEA Was More Informative Than Preoperative CEA

**Figure 1:**
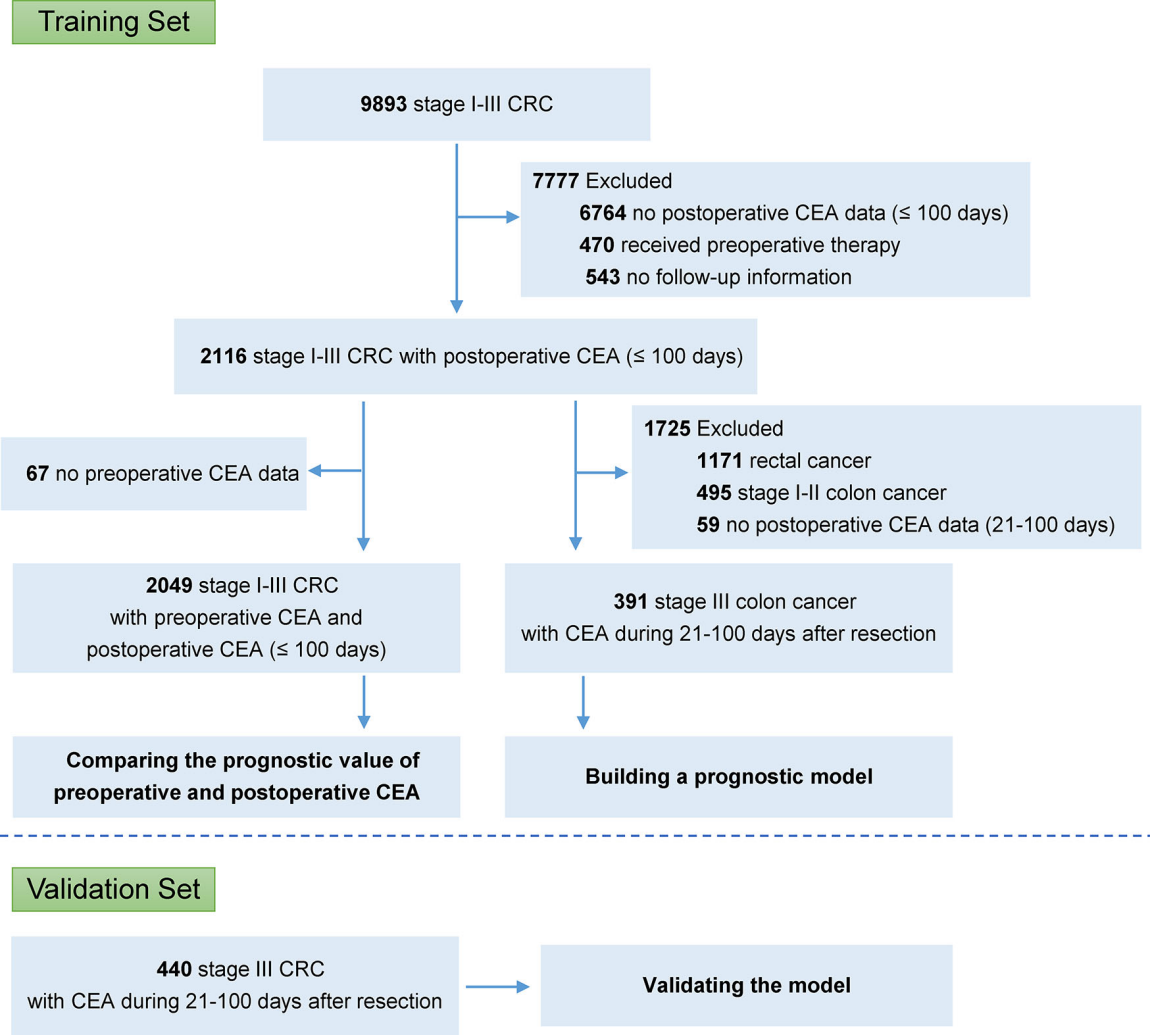
Study design.

**Figure 2:**
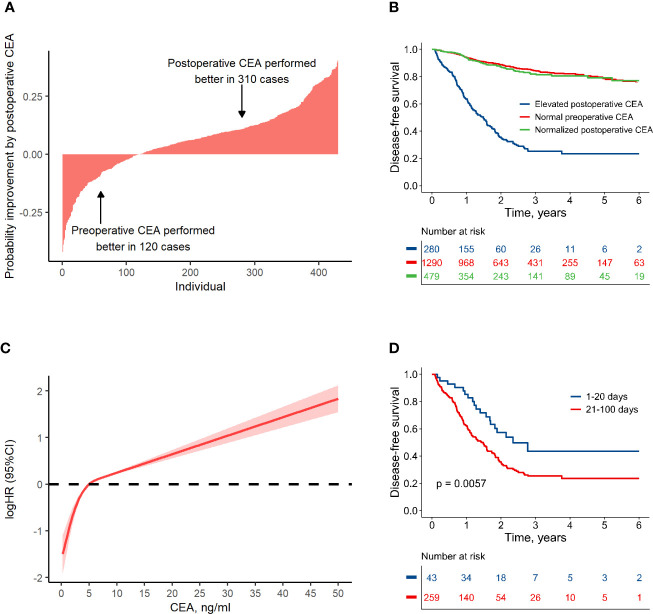
Association between colon cancer prognosis and carcinoembryonic antigen (CEA). **(A)** Individual-based probability improvement of postoperative CEA compared to preoperative CEA. This analysis was conducted in the patients whose CEA value had changed more than 2 ng/ml. **(B)** Disease-free survival by preoperative and postoperative CEA level. **(C)** The non-linear relationship pattern between hazard ratio and postoperative CEA value. **(D)** Comparison of the disease-free survival in groups with elevated postoperative CEA detected at different periods after resection.

In the FUSCC database, 2,116 patients with stage I–III colorectal cancer, who did not receive preoperative chemotherapy or radiotherapy, had CEA data within 100 days after resection (**Figure 1**; **Supplementary Table S1**). Among these patients, 2,049 also had CEA records before resection. It was shown that the predictive probabilities were improved by postoperative CEA in a large proportion of the patients when compared to preoperative CEA (**Figure 2A**). This result was validated using dichotomization process (cutoff point, 5.0 ng/ml, which was widely used) and the population was divided into three groups: (1) patients with normal (≤ 5.0 ng/ml) preoperative and postoperative CEA (normal preoperative CEA group); (2) patients with elevated (> 5.0 ng/ml) preoperative CEA but normal postoperative CEA (normalized postoperative group); and (3) patients whose preoperative and postoperative CEA levels were both elevated (elevated postoperative group). Survival analysis showed that DFS in the normalized postoperative CEA group differed little from that in the normal preoperative CEA group (HR, 1.003; 95% CI, 0.754 to 1.335; P = 0.980) (**Figure 2B**), indicating that preoperative CEA had little prognostic value in the population with normal CEA levels after resection. Therefore, the postoperative CEA was considered more informative than the preoperative CEA.

### Using RCS Method to Analyze Postoperative CEA

A non-linear relationship between postoperative CEA and logHR was shown according to RCS method (non-linear test, P < 0.001). It was worth noting that the value of 5 ng/ml was an important turning point (**Figure 2C**). For CEA < 5 ng/ml, the logHR increased rapidly with a near-linear pattern; for CEA > 5 ng/ml, the logHR increased slowly with also a near-linear pattern. Considering this non-linear relationship, the RCS method was used to process the postoperative CEA in this research.

### Optimal Time for Postoperative CEA Assessment

It takes several weeks for CEA to normalize after resection because of its half-life (16, 17). Therefore, we investigated the relationship between HR and the measure time for postoperative CEA. After adjusting CEA value as a covariate, the HR was much lower in cases who measured CEA within 20 days after surgery (**Supplementary Figure S1**). This finding was validated by dividing population into several groups with different measure time for CEA, and it was shown that among the patients with an elevated CEA measurement 1–20 days after resection, the 3-year DFS was relatively higher (approximately, 44%) than that for other time periods (approximately 25%, **Supplementary Figure S2**). Therefore, patients with elevated CEA within 100 days after resection were divided into two groups: those with an elevated CEA within 20 days after resection (early test group) and those with an elevated CEA at 21–100 days after resection (delayed test group). There were no significant differences in other clinical factors between the two groups (**Supplementary Table S2**). However, DFS in the early test group was significantly higher than that in the delayed test group (HR, 0.52; 95% CI, 0.32 to 0.83; P = 0.006) (**Figure 2D**). The risk of disease failure in the early test group was relatively low, and may have been attributable to the long half-life of CEA. To address the potential influence of false-positives, CEA data measured in first 20 days after surgery was discarded in subsequent analyses.

### The Performance of Clinical Parameters and Potential Models

A total of 391 cases of stage III colon cancer with CEA data from 21 to 100 postoperative days after resection at the FUSCC (213 males, 54.5%) were used as the training set to develop the prognostic model. The median postoperative CEA value was 2.26 ng/ml (IQR, 1.39 to 3.93), and the median postoperative CEA measured time after resection was 35 (IQR, 27 to 84) days. The median follow-up duration was 28 months (IQR, 14 to 44). A total of 82 patients (21.0%) had disease failure, and the 3-year DFS rate for all patients was 73.0%. Further validation was conducted in the dataset with 440 cases of stage III colon cancer from 2007 to 2017 from Liaoning Cancer Hospital and Harbin Medical University Cancer Hospital, and the clinicopathological characteristics of these patients were similar to those in the training set (**Table 1**).

**Table 1:** Baseline characteristics of training and validations sets.

Variables	Training set	Validation set
Total	391	440
Sex (%)		
Female	178 (45.5)	197 (44.7)
Male	213 (54.5)	244 (55.3)
Age		
Median(IQR)	59 (50–66)	59 (52–65)
T stage (%)		
T1–T2	19 (4.8)	18 (4.0)
T3	132 (33.8)	221 (50.1)
T4	240 (61.4)	202 (45.8)
N stage (%)		
N1a	84 (21.5)	157 (35.6)
N1b	105 (26.9)	147 (33.3)
N1c	38 (9.7)	4 (0.9)
N2a	86 (22.0)	75 (17.0)
N2b	78 (19.9)	58 (13.2)
Positive lymph nodes		
Median (IQR)	2 (1–4)	2 (1–4)
Postoperative CEA (%)		
Median(IQR), ng/ml	2.26 (1.39–3.93)	1.79 (1.13–2.82)
≤5 ng/ml	319 (81.6)	398 (90.2)
>5 ng/ml	72 (18.4)	43 (9.7)
Postoperative CA19-9 (%)		
Median(IQR), U/ml	11.1 (6.50–19.48)	10.64 (6.82–18.71)
≤37 U/ml	346 (88.5)	395 (89.6)
>37 U/ml	42 (10.7)	26 (5.9)
Unknown	3 (0.7)	20 (4.5)
Perineural invasion (%)		
Negative	281 (71.9)	377 (85.5)
Positive	108 (27.6)	46 (10.4)
Unknown	2 (0.5)	18 (4.0)
Lymphovascular invasion (%)		
Negative	218 (55.8)	369 (83.7)
Positive	168 (43.0)	55 (12.5)
Unknown	5 (1.2)	17 (3.8)
Differentiation (%)		
Low	119 (30.4)	96 (21.8)
Middle	241 (61.6)	309 (70.1)
High	15 (3.8)	9 (2.0)
Unknown	16 (4.0)	27 (6.1)
Site (%)		
Left	193 (49.4)	246 (55.8)
Right	198 (50.6)	195 (44.2)

CA19-9, carbohydrate antigen 19-9; CEA, carcinoembryonic antigen; IQR, interquartile range; RCS, restricted cubic splines.

**Figure 3:**
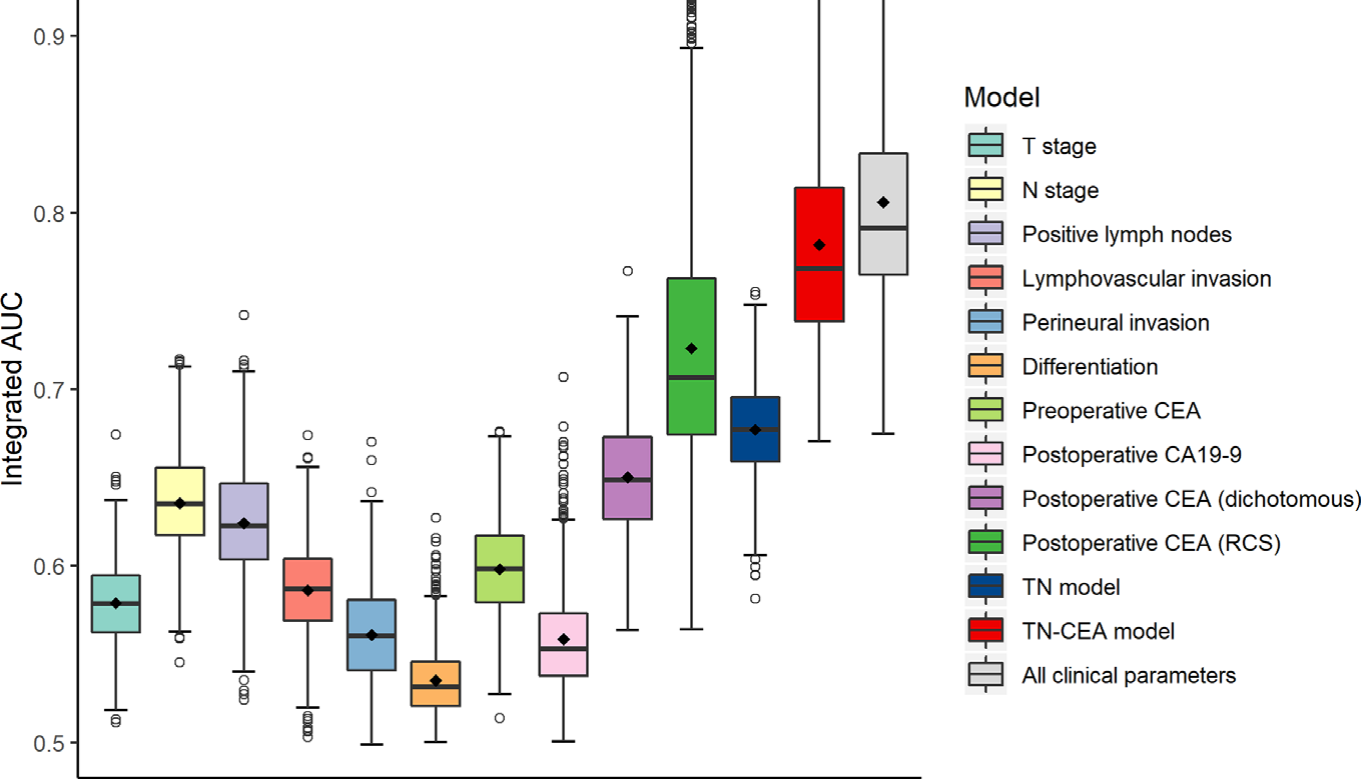
Clinical performance of tumor-related risk parameters. The predictive accuracy for disease-free survival based on the iAUC with 1,000 × bootstrap resampling for each parameter is shown as a box plot. The iAUC indicates integrated area under the ROC curve.

To build the prognostic model, the clinical variables including T stage, N stage, postoperative CEA, postoperative carbohydrate antigen 19-9 (CA19-9), perineural invasion, lymphovascular invasion, and differentiation were considered. Univariate and multivariate analyses for DFS were performed using a Cox proportional hazards model; the CEA value was processed using dichotomization and RCS, respectively (**Table 2**). Multivariate analysis showed that T stage, N stage, and postoperative CEA were independent prognostic variables (population with elevated postoperative CEA vs. normal postoperative CEA, HR, 4.08; 95% CI, 2.47 to 6.74; *P* < 0.001; RCS: *P* < 0.001). The predictive accuracy for DFS based on the iAUC with 1,000 × bootstrap resampling for the parameters was shown as a box plot in **Figure 3**. Among the univariate models, the postoperative CEA (processed with RCS) had the highest mean iAUC (0.72). Although both were univariate predictors of postoperative CEA, the RCS-based method is significantly better than the dichotomization-based method (*P* < 0.001).

**Table 2:** Univariate and multivariate analysis.

Variables	Univariate Analysis		Multivariate Analysis			
			Dichotomization		RCS	
	HR (95% CI)	*P* value	HR (95% CI)	*P* value	HR (95% CI)	*P* value
T stage		0.007		0.021		0.013
T4	1 (Ref)	–	1 (Ref)	–	1 (Ref)	–
T3	0.52 (0.29–0.92)	0.025	0.51 (0.29–0.91)	0.022	0.48 (0.26–0.88)	0.018
T1-2	0.18 (0.02–1.29)	0.088	0.27 (0.03–1.97)	0.197	0.24 (0.03–1.76)	0.161
N stage		0.002		0.022		0.035
N2b	1 (Ref)	–	1 (Ref)	–	1 (Ref)	–
N2a	0.87 (0.50–1.52)	0.626	0.88 (0.49–1.57)	0.679	0.99 (0.55–1.77)	0.972
N1c	0.25 (0.08–0.73)	0.011	0.26 (0.08–0.76)	0.015	0.30 (0.10–0.91)	0.034
N1b	0.42 (0.22–0.79)	0.008	0.50 (0.25–0.98)	0.044	0.51 (0.26–1.03)	0.062
N1a	0.38 (0.19–0.77)	0.007	0.48 (0.22–1.02)	0.057	0.48 (0.22–1.04)	0.062
Postoperative CEA		<0.001		<0.001		–
≤5 ng/ml	1 (Ref)	–	1 (Ref)	–	–	–
>5 ng/ml	5.53 (3.54–8.63)	<0.001	4.08 (2.47–6.74)	<0.001	–	–
Postoperative CEA (RCS)		<0.001		–		<0.001
Linear	–	<0.001	–	–	–	<0.001
Non-linear	–	<0.001	–	–	–	<0.001
Postoperative CA19-9		<0.001		0.315		0.171
≤37 U/ml	1 (Ref)	–	1 (Ref)	–	1 (Ref)	–
>37 U/ml	2.79 (1.59–4.92)	<0.001	1.38 (0.73–2.57)	0.315	1.54 (0.83–2.85)	0.171
Postoperative CA19-9 (RCS)		0.106		–		–
Linear	–	0.179	–	–	–	–
Non-linear	–	0.210	–	–	–	–
Perineural invasion		0.021		0.138		0.255
Negative	1 (Ref)	–	1 (Ref)	–	1 (Ref)	–
Positive	1.70 (1.08–2.68)	0.021	1.44 (0.88–2.34)	0.138	1.33 (0.81–2.16)	0.255
Lymphovascular invasion		0.004		0.495		0.383
Negative	1 (Ref)	–	1 (Ref)	–	1 (Ref)	–
Positive	1.90 (1.23–2.94)	0.004	1.18 (0.72–1.93)	0.495	1.25 (0.76–2.04)	0.383
Differentiation		0.571		–		–
Low	1 (Ref)	–	–	–	–	–
Middle	0.89 (0.55–1.46)	0.655	–	–	–	–
High	0.49 (0.11–2.11)	0.344	–	–	–	–

Multivariate analysis was performed using dichotomization and RCS for postoperative CEA, respectively.

CA19-9, carbohydrate antigen 19-9; CEA, carcinoembryonic antigen; CI, confidence interval; HR, hazard ratio; Ref, reference; RCS, restricted cubic splines.

### The Optimal Prognostic Model for Stage III Colon Cancer

**Figure 4:**
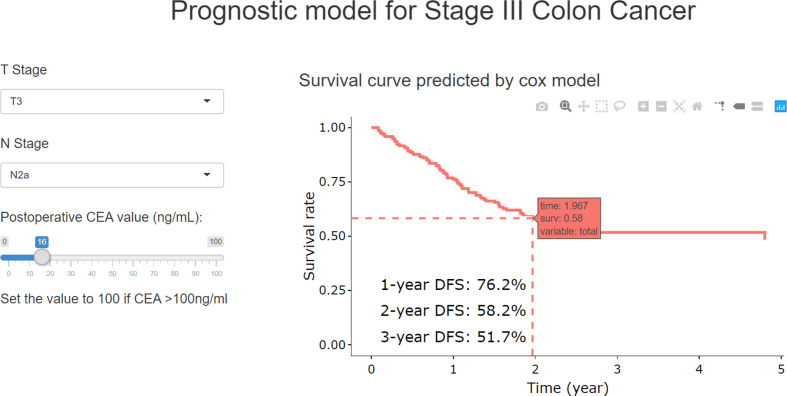
The online web-tool for the TN-CEA model.

With RCS process, the postoperative CEA was combined with T and N stage to build the final model (TN-CEA model). A web tool was developed based on this TN-CEA model (**Figure 4**; http://123.206.185.159:6060/, the main site; or https://fan-app.shinyapps.io/zhulab-coloncancer/, the alternate site), which was more convenient than nomogram. With this tool, users no longer need to calculate survival rate by themselves and could interactively access the predicted survival curve for the concerned patient. Of course, the nomogram was also still available (**Supplementary Figure S3A**), and the calibration curves showed in **Supplementary Figure S4**. Data distribution was shown in **Supplementary Figure S5**, which was sorted with increasing predicted-risk score. To confirm whether the N stage could be further optimized, we also assessed the similar model (**Supplementary Figure S3B**), which consisted of T stage, number of positive lymph nodes, and postoperative CEA, and found out that the performance of this model was close to TN-CEA model.

**Figure 5:**
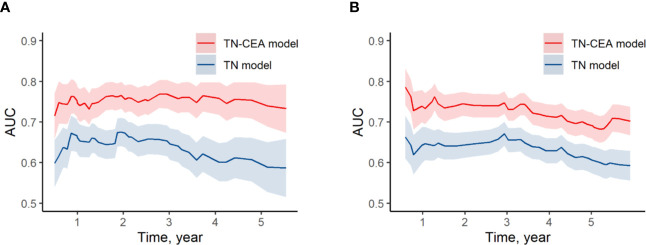
Time-dependent AUC of models. **(A)** Time-dependent AUC of TN model and TN-CEA model in training set. **(B)** Time-dependent AUC of TN model and TN-CEA model in validation set. TN model, the model includes the T stage and the N stage. TN-CEA model, the model includes T stage, N stage, and postoperative CEA.

When the postoperative CEA was added to the model consisting of T and N stages (mean iAUC, 0.66), the performance of the new model was significantly improved (likelihood ratio *P* < 0.001), and the mean iAUC of the TN-CEA model reached to 0.78 (**Figure 5A**, **Supplementary Figure S6**). This improvement was also confirmed in the validation set (iAUC for TN model vs. TN-CEA model: 0.63 vs. 0.73; **Figure 5B**). When compared with the TN model, the TN-CEA model achieved about 15% NRI (**Supplementary Figure S7**; the TN-CEA model also achieved over 10% NRI in the validation set), and the DCA also supported these results (**Supplementary Figure S8**). In the TN-CEA model, the relative contribution of postoperative CEA was greater than that of the other two variables (**Supplementary Figure S9**).

**Figure 6:**
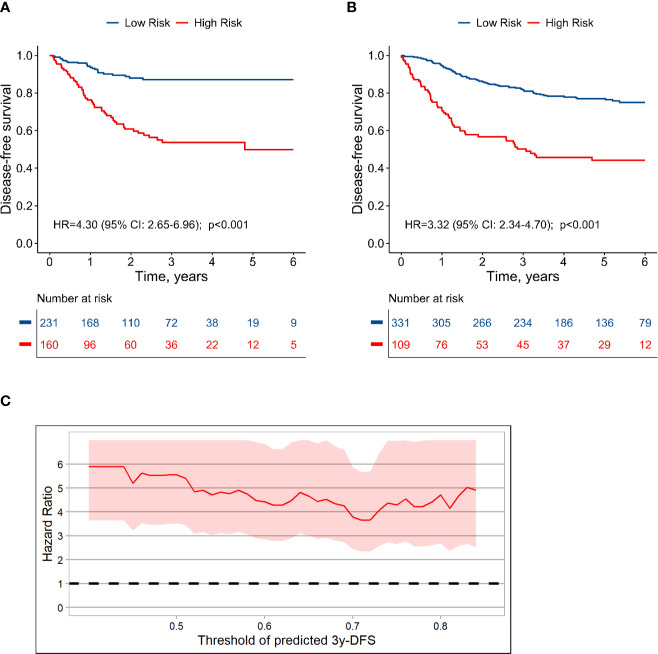
Dividing stage III colon cancer into high and low risk groups based on the TN-CEA model. **(A)** Disease-free survival in the training dataset. **(B)** Disease-free survival in the validation dataset. **(C)** Hazard ratio for high-risk versus low-risk group at different thresholds. This analysis was based on the predicted 3-year disease-free survival rate.

The population could be divided into high risk group (160 cases; predicted 3-year DFS: ≤ 75%) and low risk group (231 cases; predicted 3-year DFS: >75%), and the actual 3-year DFS for high-risk and low-risk groups were 53.7% and 87.0%, respectively (HR, 4.30; 95% CI, 2.65 to 6.96; *P* < 0.001) (**Figure 6A**). In the validation set, the 3-year DFS values for the high-risk and low-risk groups were 50.2% and 81.6% (HR, 3.32; 95% CI, 2.34 to 4.70; *P* < 0.001), respectively (**Figure 6B**). CEA measured before or during adjuvant therapy had no significant effect on this stratification (**Supplementary Figure S10**; interaction test: *P* > 0.1). In the sub-population with CEA before adjuvant, HR for high-risk group vs. low-risk group was 3.5 (*P* < 0.001). Moreover, using other series cutoff-points besides 75% as threshold for high and low risk could also generate significant stratification with HR value around 4.0 (**Figure 6C**, **Supplementary Figure S11**), which indicated that the patients with stage III colon cancer could be well stratified based on the TN-CEA model.

## Discussion

Following the publication of the IDEA study report, the need for stratified treatment for stage III colon cancer has received global recognition (2). However, the debate on how to distinguish between high-risk and low-risk groups is ongoing, given the limitations of the TNM system for staging colon cancer. The feasibility of conducting a prospective study in > 10,000 patients, such as the IDEA study, is limited. It is therefore important to retrospectively search for potential biomarkers and verify their prognostic value. Therefore, we conducted this retrospective study and found that the postoperative CEA was an extraordinarily valuable prognostic factor for colon cancer and significantly improved the performance of the TNM model. When postoperative CEA was integrated into the prognostic model, a strong discriminatory ability was demonstrated: the integrated-AUC of the model reached 0.78. In addition, our model appeared to be robust and reproducible in the validation dataset from the Liaoning Cancer Hospital and Harbin Medical University Cancer Hospital. Patients with stage III colon cancer are a subgroup selected based on the use of prognostic model, and it is not easy to further stratify the prognosis in this subset population. The new model from this research offered a possibility to generate a significantly better solution: patients could be obviously divided into high and low risk groups using this model and the given threshold.

CEA has been established as an attractive prognostic variable for colorectal cancer. Previous studies have shown that elevated preoperative CEA levels represent an independent risk factor (3–5, 18–20). Additionally, failure to normalize CEA levels after resection has been shown to play an important role in poor prognosis (21–25). Recently, a retrospective study of 1,027 patients with stage I–III colon cancer conducted by the Memorial Sloan Kettering Cancer Center showed that patients with elevated preoperative CEA and normalized after resection, had recurrence-free survival similar to that of patients with normal preoperative CEA, which indicated that elevated postoperative CEA was a more important indicator for prognosis in colon cancer than preoperative CEA (6). However, the investigators did not define the timeframe for postoperative CEA detection. Considering the half-life of CEA in the blood, it can be difficult to avoid false-positive results if the CEA testing time is too close to resection. Our analysis showed that the patients with elevated CEA within 20 days after resection had a significantly better prognosis compared to the patients with elevated postoperative CEA measured at delayed time. Therefore, the first 20 days after resection was not recommended to assess the CEA. Additionally, processing CEA as dichotomous variable (normal vs. elevated) would lose the real relationship pattern between CEA and survival. Instead, we recommend the RCS method to process CEA value. Other potential risk factors such as preoperative CEA, perineural invasion, lymphatic/vascular invasion, and differentiation were also analyzed in a multivariate model. These variables had a lower impact than postoperative CEA, indicating that the latter was more influential in the prognosis of colon cancer.

In addition to serum CEA, other potential prognostic biomarkers have previously been investigated to identify a more effective classification for colon cancer (26–28). In a pooled analysis for stage II–III colon cancer, microsatellite instability, and *KRAS* and *BRAF* mutation status were used to construct a prognostic model in combination with TNM stage. C-index values increased from 0.61–0.68 to 0.63–0.71 when combined with the molecular markers above, indicating that these molecular biomarkers marginally improved prognostic performance, but at a significantly higher cost than CEA (29). Another potential indicator of interest is the Immunoscore, which has been widely paid attention in colon cancer. Within a prognostic model, the Immunoscore has been shown to significantly improve the model performance (30). However, the complexity of measuring Immunoscore limits its use in clinical practice. Additionally, some studies have explored the prognostic value of circulating tumor DNA (ctDNA) in colorectal cancer (31–35). However, no large-scale study of ctDNA or comprehensive evaluation of a prognostic stratification model containing ctDNA has been reported to date. Some researchers believed that ctDNA was better than CEA (36). However, the reported data always included the CEA tested within 20 days after surgery which was not suitable for assessment due to the false-positives, and those research processed CEA as dichotomous variable which underestimated the potential of CEA. In addition, ctDNA detection was so expensive that it was hard to promote ctDNA as a routine test for colon cancer especially in developing countries. Compared with these candidate prognostic biomarkers, postoperative CEA is more accurate, cost-effective, and widely available, and has been used routinely as a test for colorectal cancer for some decades. Furthermore, this TN-CEA model consisted of only three variables, and more variables could be carefully selected and combined into this model in the future. To integrate multi-omics and clinical data, a more comprehensive research based on this model is ongoing in our center.

In terms of practicality, we believe that our findings provide evidence for the individualized treatment of patients with stage III colon cancer meaning that, for example, low-risk patients could avoid overtreatment while those classified as high risk could maintain the intensity of current adjuvant therapy. For patients at critical risk, further investigation may be warranted to determine whether to increase maintenance with a single agent after 6 months of standard adjuvant chemotherapy with FOLFOX/XELOX.

There are limitations to this exploratory study. Firstly, given the retrospective study design, we did not formulate a standard for the entire course of treatment, particularly for the adjuvant course. To avoid bias induced by inappropriate treatment, three leading colorectal cancer centers in China, at which patients accepted a standardized treatment plan according to national guidelines, were invited to participate in our study. Secondly, the 391 patients in training set were selected from a pool of 1,893 patients with stage III colon cancer in total, due to many patients had lacked postoperative CEA record or missing follow-up information in this retrospective research. Thirdly, the timeframe of CEA testing was defined as 21–100 days after radical surgery, which may have affected the accuracy of postoperative CEA for timeframes longer than 2 months. The choice of timeframe was primarily based on the following considerations: false-positive CEA results occur more frequently within 20 days after surgery, which was confirmed in our study; the upper limit of 100 days was selected because the decision whether to continue adjuvant chemotherapy is typically made at approximately that time-point. Fourthly, the sequence of CEA test and adjuvant chemotherapy has not been fully considered in this study.

## Conclusion

In summary, the stage III colon cancer population is heterogeneous with diverse prognoses, meaning that a uniform treatment mode is no longer applicable to all patients. These patients could be stratified much more accurately with the prognostic model incorporating postoperative CEA, T, and N stages, and our model may provide evidence for individualized postoperative adjuvant chemotherapy regimens.

## Data Availability Statement

The raw data supporting the conclusions of this article will be made available by the authors, without undue reservation.

## Ethics Statement

The studies involving human participants were reviewed and approved by the Institutional Review Board at Fudan University Shanghai Cancer Center. The ethics committee waived the requirement of written informed consent for participation.

## Author Contributions

JZ initiated and coordinated this study. JZ, RZ, YL, and JF designed the study. JF, YL, XC, JW, RG, CL, YX, and XL collected the data. JF, JZ, and YJ analyzed the data. JF, CZ, and JZ wrote the manuscript. JZ, CZ, RZ, YL, XC, and YJ revised the manuscript. SC supervised the study. All authors contributed to the article and approved the submitted version.

## Funding

This research was funded, in part, by Natural Science Foundation of Shanghai, grant number: 19ZR1410600; Shenyang Science and Technology Plan Population and Health Applied Technology Research Project, grant number: 18-014-4-02; Harbin Medical University Cancer Hospital Preeminence Youth Fund, grant number: JCQN2019-04; and the China Scholarship Council, grant number: 201908050148. The funders had no role in the design and conduct of the study; collection, management, analysis, and interpretation of the data; preparation, review, or approval of the manuscript; and decision to submit the manuscript for publication.

## Conflict of Interest

The authors declare that the research was conducted in the absence of any commercial or financial relationships that could be construed as a potential conflict of interest.
