# Supplementary material for: A Novel Prognostic Model Incorporating Carcinoembryonic Antigen in 3-Week or Longer Postoperative Period for Stage III Colon Cancer: A Multicenter Retrospective Study
Source: Front Oncol. 2020 Dec 1;10:566784. doi: 10.3389/fonc.2020.566784 (PMC7736239; doi:10.3389/fonc.2020.566784)
Supplement: Supplementary file 1 [file DataSheet_1.pdf]

Supplementary Material

Contents

Supplementary Figures

Supplementary Figure S1. The relationship between Hazard ratio and the time for postoperative CEA measurement.

Supplementary Figure S2. Comparison of the DFS of 4 groups with elevated postoperative CEA detected in different periods after resection.

Supplementary Figure S3. The nomogram of the models.

Supplementary Figure S4. Calibration curves.

Supplementary Figure S5. Data distribution of variables and outcome for each patient.

Supplementary Figure S6. ROC for TN-CEA model at specific time points.

Supplementary Figure S7. Net reclassification improvement.

Supplementary Figure S8. Decision curve analysis.

Supplementary Figure S9. Relative importance of each risk parameter for survival risk.

Supplementary Figure S10. Disease-free survival of high-risk group and low-risk group in the patients with CEA measured before or during adjuvant.

Supplementary Figure S11. Hazard ratio for high-risk versus low-risk group at different thresholds in validation dataset.

Supplementary Tables

Supplementary Table S1. Basic characteristics of patients with stage I-III stage colon cancer in FUSCC datasets.

Supplementary Table S2. Comparison of characteristics of two groups with elevated postoperative CEA detected in different periods after resection.

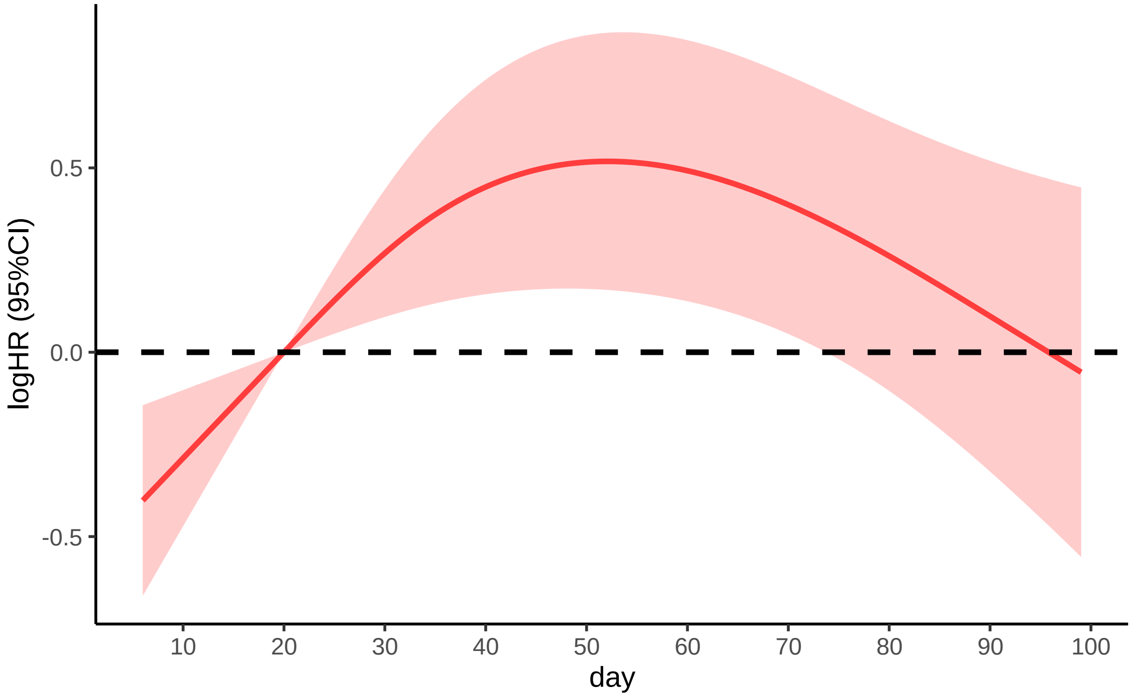

Supplementary Figure S1. The relationship between hazard ratio (HR) and the time for postoperative CEA measurement. The relationship between HR and the time for postoperative CEA measurement was analyzed using restricted cubic splines, and the result was adjusted by postoperative CEA value.

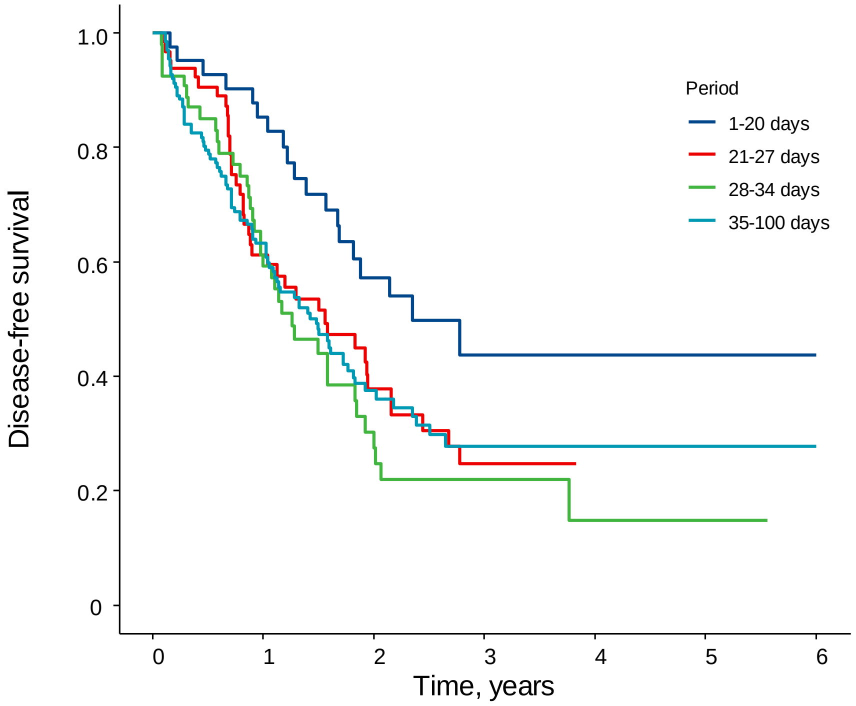

No. at risk

	0	1	2	3	4	5	6
1-20 days	43	34	18	7	5	3	2
21-27 days	66	34	16	7	0	0	0
28-34 days	54	31	11	6	2	1	0
35-100 days	139	75	27	13	8	4	1

Supplementary Figure S2. Comparison of the DFS of 4 groups with elevated postoperative CEA detected in different periods after resection.

A

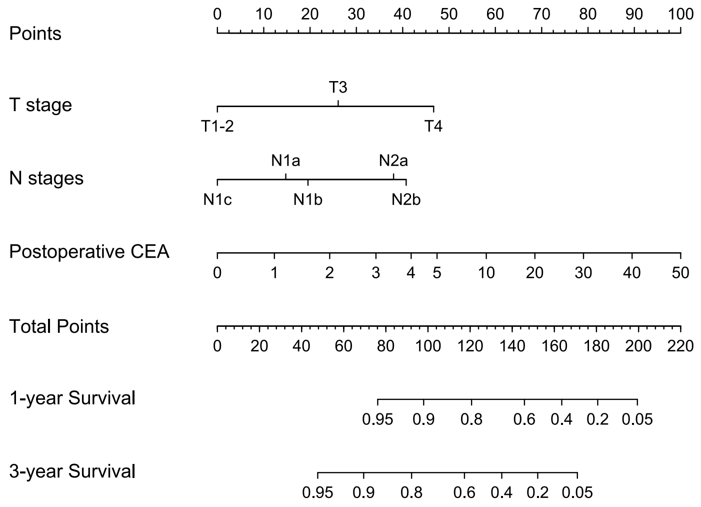

B

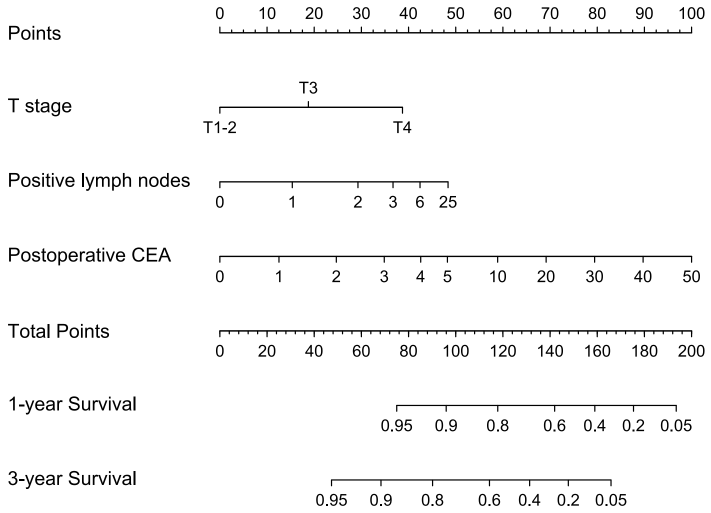

Supplementary Figure S3. The nomogram of the models. (A) The nomogram of the TN-CEA model. (B) The nomogram of the model consisted of T stage, number of positive lymph nodes, and postoperative CEA. These two models could be replaced with each other as the performances of them were similar.

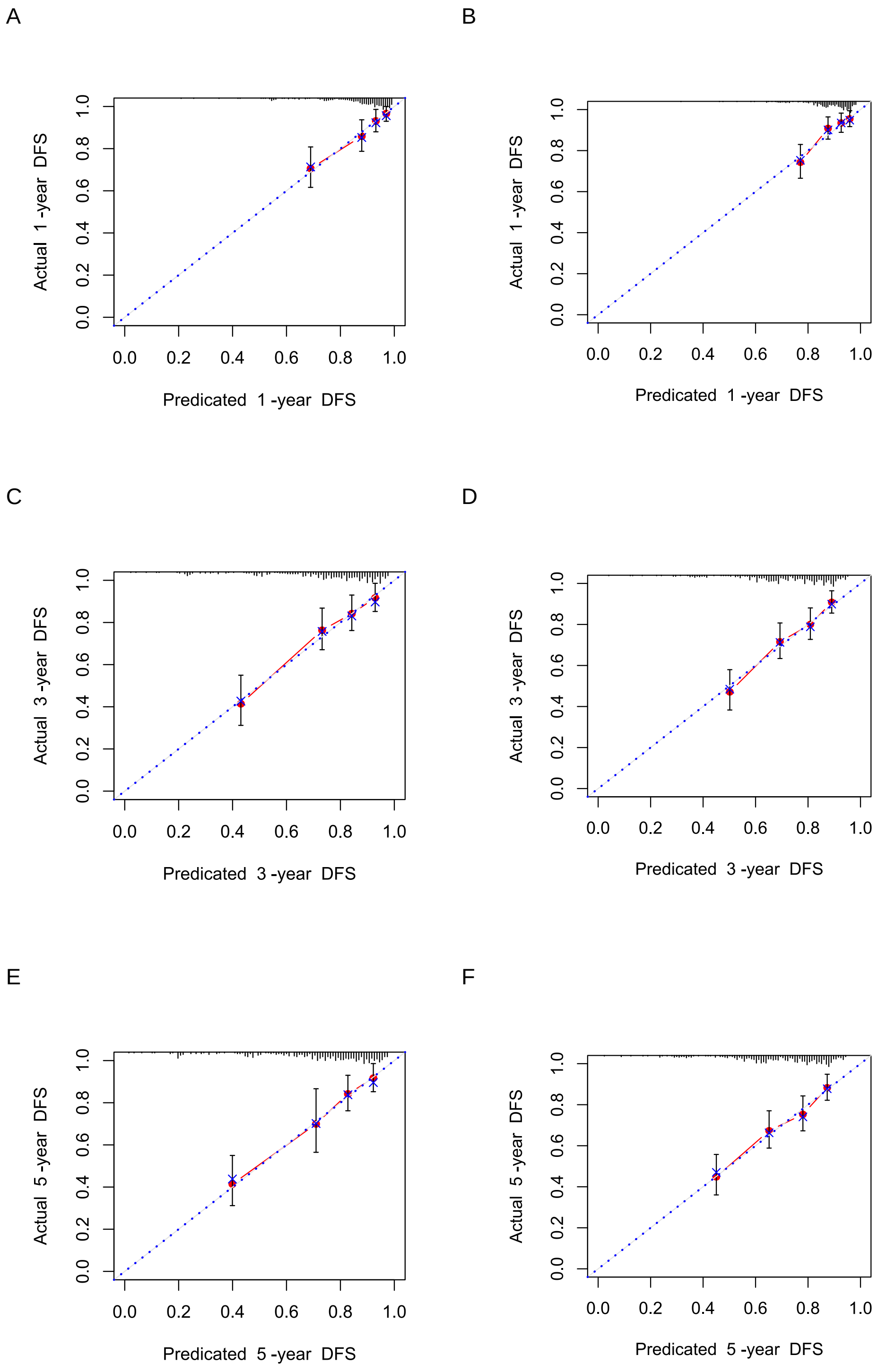

Supplementary Figure S4. Calibration curves. The calibration curve for 1-year, 3-year and 5-year in training dataset were shown as (A), (C) and (E); the calibration curve for 1-year, 3-year and 5-year in validation dataset were shown as (B), (D) and (F).

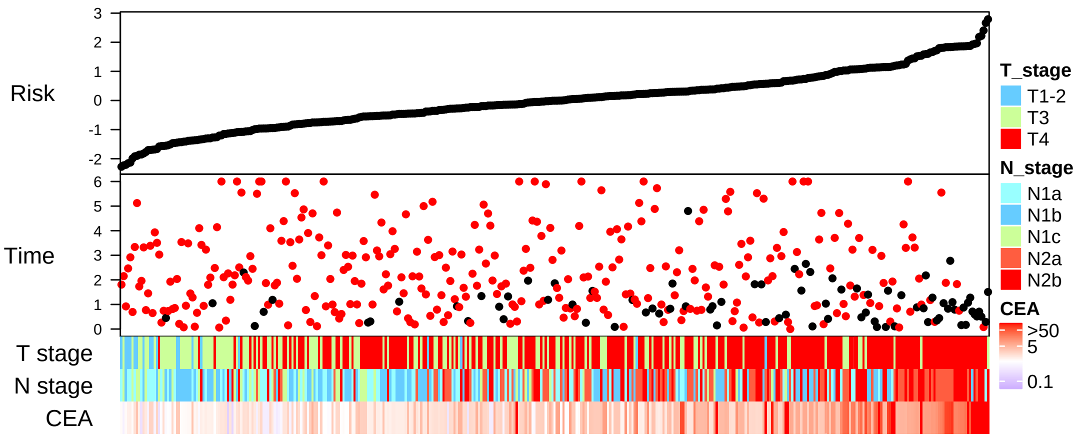

Supplementary Figure S5. Data distribution of variables and outcome for each patient. The patients were arranged in order of increasing risk predicted by TN-CEA model. Dot in red indicated censor, and in black indicated disease recurrence or dead.

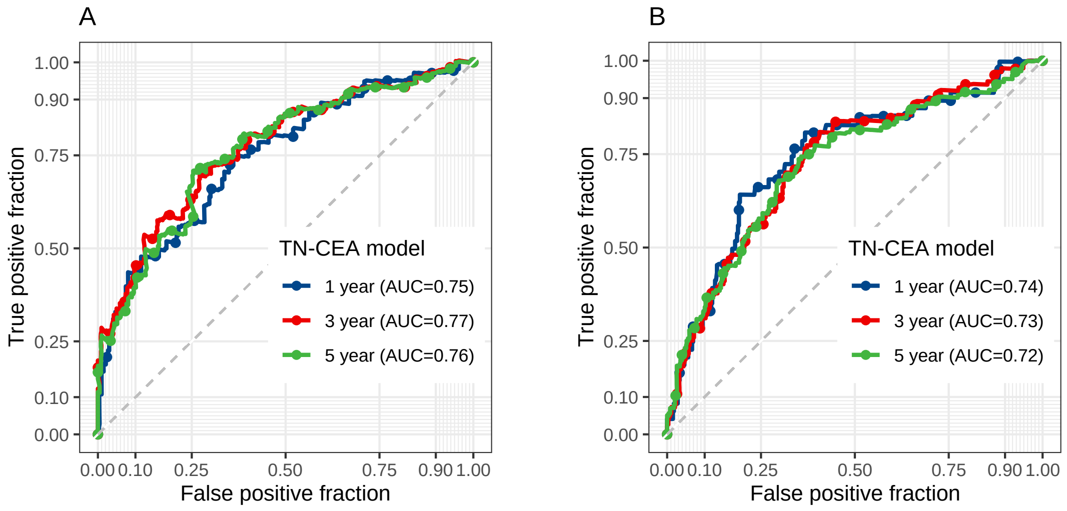

Supplementary Figure S6. ROC for TN-CEA model at specific time points. (A) ROC for 1-year, 3-year and 5-year in training dataset; (B) ROC for 1-year, 3-year and 5-year in validation dataset.

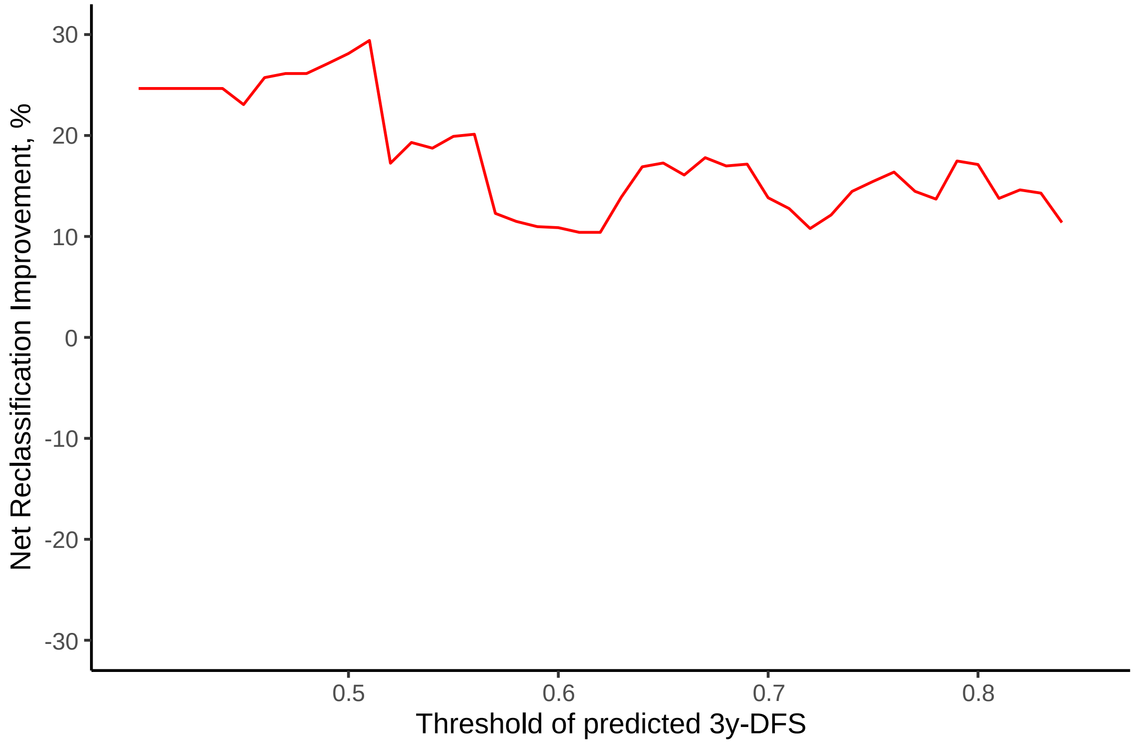

Supplementary Figure S7. Net reclassification improvement.

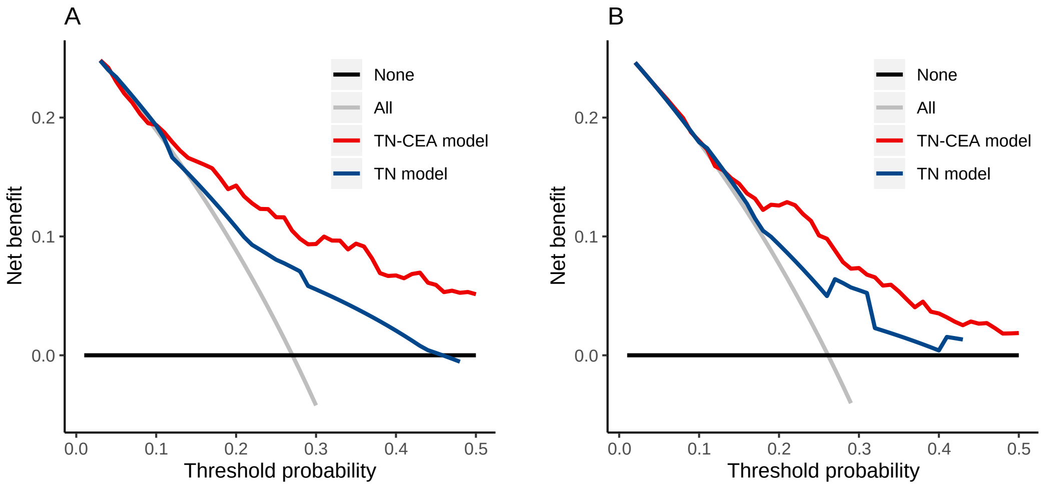

Supplementary Figure S8. Decision curve analysis. (A) Decision curve analysis in training dataset; (B) Decision curve analysis in validation dataset.

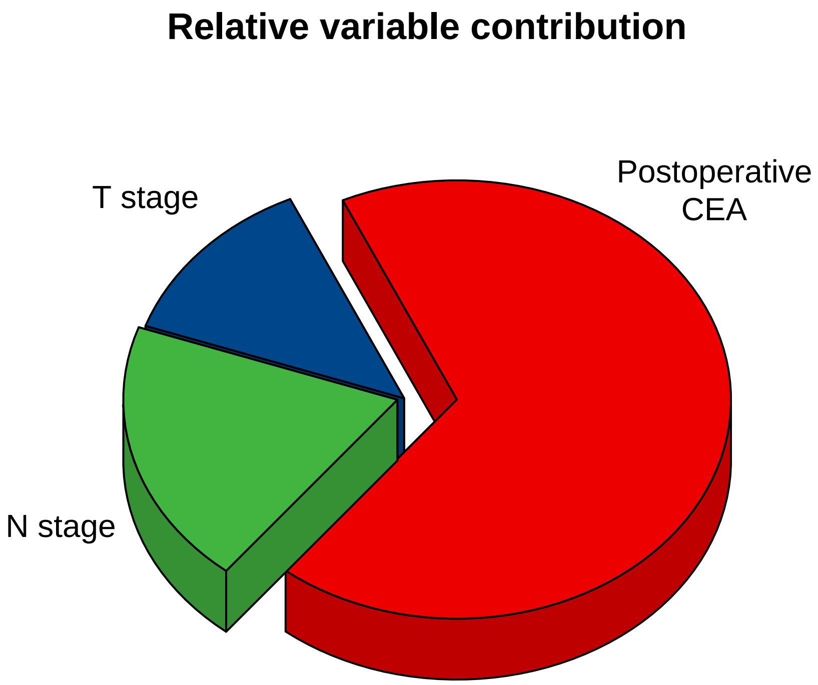

Supplementary Figure S9. Relative importance of each risk parameter for survival risk. The relative importance was calculated by using the χ^2 value from Harrell's rms R package.

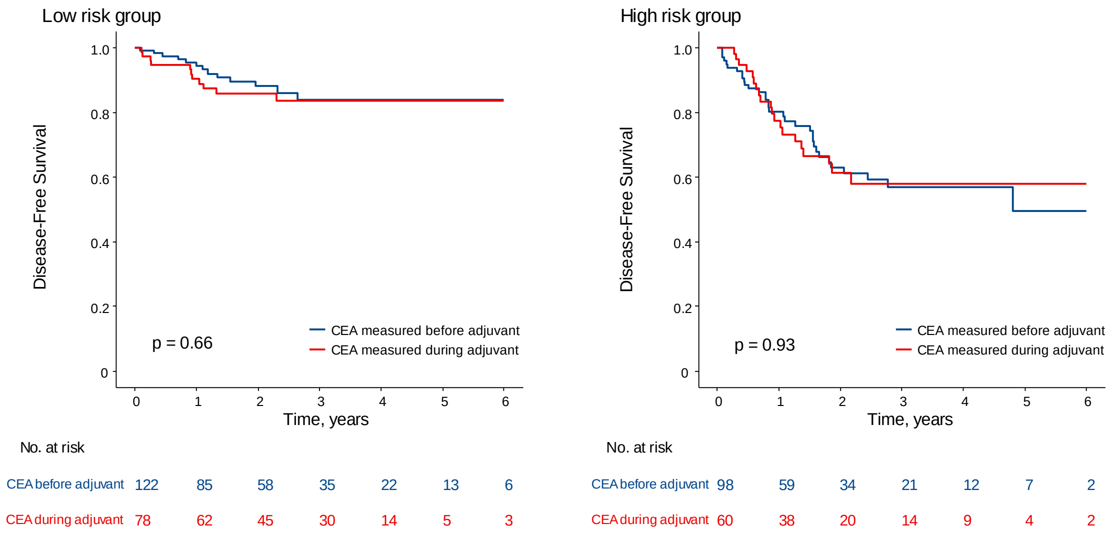

Supplementary Figure S10. Disease-free survival of high-risk group and low-risk group in the patients with CEA measured before or during adjuvant. 33 cases whose adjuvant time was unsure were not included in this subgroup analysis.

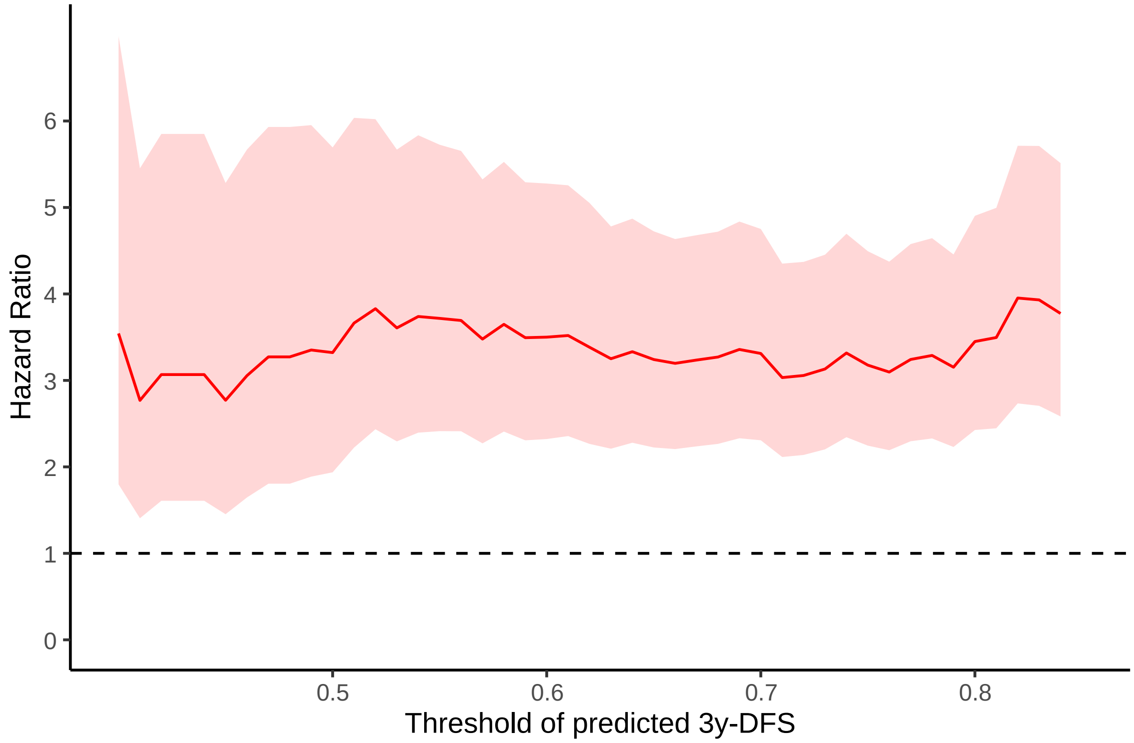

Supplementary Figure S11. Hazard ratio for high-risk versus low-risk group at different thresholds in validation dataset.

Supplementary Table S1. Basic characteristics of patients with stage I-III stage colon cancer in FUSCC datasets.

	Patients with postoperative CEA[b]	All patients
Total, No.	2116	9893
Sex, No.(%)		
Male	1234 (58.3)	5895 (59.6)
Female	882 (41.7)	3998 (40.4)
Age, median(IQR), y	58 (50-65)	59 (51-66)
T stage, No.(%)		
T0	29 (1.4)	237 (2.4)
T1	120 (5.7)	587 (5.9)
T2	328 (15.5)	1619 (16.4)
T3	760 (35.9)	3361 (34.0)
T4	851 (40.2)	3634 (36.7)
Unknown	28 (1.3)	455 (4.6)
N stage, No.(%)		
N0	1020 (48.2)	5322 (53.8)
N1	591 (27.9)	2525 (25.5)
N2	505 (23.8)	2046 (20.7)
Preoperative CEA >5 ng/ml, No.(%)[a]	729 (34.5)	3183 (32.2)
Postoperative CEA >5 ng/ml, No.(%)	302 (14.3)	-
Follow-up, median(IQR), month	29 (15-47)	27 (14-48)
3-year DFS	75.4%	75.8%

[a]Preoperative CEA was not available for 67 cases and 737 case, respectively. [b]The period for postoperative CEA was restricted within 100 days after resection in this table.

Supplementary Table S2. Comparison of characteristics of two groups with elevated postoperative CEA detected in different periods after resection.

	1-20 days	21-100 days	
	Patients, No. (%)	Patients, No. (%)	P value[a]
T stage			0.10
T1-2	5 (11.6)	19 (7.3)	
T3	17 (39.5)	73 (28.2)	
T4	20 (46.5)	166 (64.1)	
Unknown	1 (2.3)	1 (0.3)	
N stage			0.68
N0	11 (25.6)	63 (24.3)	
N1	16 (37.2)	82 (31.7)	
N2	16 (37.2)	114 (44.0)	
Perineural invasion			1.00
Negative	29 (67.4)	175 (67.6)	
Positive	13 (30.2)	82 (31.7)	
Unknown	1 (2.3)	2 (0.8)	
Lymphovascular invasion			1.00
Negative	23 (53.5)	136 (52.5)	
Positive	19 (44.2)	116 (44.8)	
Unknown	1 (2.3)	7 (2.7)	
Differentiation			0.85
Low	29 (67.4)	173 (66.8)	
Middle/High	11 (25.6)	75 (29.0)	
Unknown	3 (7.0)	11 (4.2)	
Preoperative CEA			0.22
⩽ 5 ng/ml	1 (2.3)	29 (11.2)	
> 5 ng/ml	31 (72.1)	219 (84.6)	
Unknown	11 (25.6)	11 (4.2)	

[a]Fisher exact test p value.
